# Clutter Subspace Characteristics-Aided Space-Time Adaptive Outlier Sample Selection Method

**DOI:** 10.3390/s21093108

**Published:** 2021-04-29

**Authors:** Dongning Fu, Guisheng Liao, Jingwei Xu

**Affiliations:** National Lab of Radar Signal Processing, School Electronic Engineering, Xidian University, Xi’an 710071, China; dongningfu@stu.xidian.edu.cn (D.F.); jwxu@xidian.edu.cn (J.X.)

**Keywords:** space-time adaptive processing, clutter subspace, sample selecting, airborne radar

## Abstract

For statistic space-time adaptive processing (STAP), a critical issue is estimating the clutter covariance matrix (CCM). However, sufficient training samples are difficult to obtain that satisfy the independent and identically distributed (IID) condition. It is because of the realistic heterogeneous environment faced by airborne radar. Moreover, one should eliminate contaminated training samples before CCM estimation. Aiming at the problems of the computational complexity and susceptibility to the outlier of the traditional generalized inner product (GIP) method, a clutter subspace-based training sampling selecting method is proposed combined with specific distribution in the space-time plane of clutter spectrum. Theoretical analysis and simulation results verified the proposed method and indicate that the proposed method is easy to construct CCM and has lower computational complexity and sensitivity to outliers.

## 1. Introduction

Estimating the time-space clutter covariance matrix (ST-CCM) is one of the most critical issues in adaptive space-time statistical processing (STAP) [1,2,3,4]. However, it is difficult to obtain sufficient training samples to satisfy the requirement for independent and identically distributed (IID). This is due to the realistic heterogeneous environment [5] that airborne radar faces. The methods for the problems of clutter heterogeneity have several major categories, such as the power selected training (PST) [6] method, the direct data domain (DDD) [7,8,9,10,11,12,13] method, non-homogeneity detection (NHD) [14,15,16,17,18] method, knowledge-aided (KA) [19,20] method and so on. Existing NHD method include: generalized inner product (GIP) [15,17,18], sampling covariance matrix inverse (SMI) [14,18,21], correlation dimension (CD) [22], cyclic detection of adaptive power residual (APR) [23,24,25,26], and geometric center of mass criterion [27,28].

The yaw angle [29,30,31,32] of the airborne system and the variable clutter environment [33,34,35,36] may account for this. Furthermore, we cannot ignore the outlier [14,21,37,38,39,40,41], which is a critical factor affecting the ST-CCM estimate’s accuracy. Assume that there are outliers in the training samples used to estimate the ST-CCM. In that case, it will happen that the mismatch of the weight vector calculation, the loss of the output signal to clutter noise ratio (SCNR), and decrease of the detection performance of low speed and weak moving targets. Therefore, before estimating the ST-CCM, it is necessary to eliminate the training samples contaminated with interference or outlier.

The primary technical basis for traditional STAP sample selection methods is the generalized inner product (GIP) [38,39,40,41]. However, the GIP reference matrix is unknown because it is impossible to obtain the accurate ST-CCM from the under-tested range samples in the practical environment. Therefore, the traditional GIP method typically uses both sides of the under-tested range sample data as training samples to estimate the GIP matrix. However, the heterogeneous clutter environment will cause the estimated GIP matrix to be seriously mismatched. The reason includes varying degrees of heterogeneity of each training sample and the influence of potential outliers’ shielding effect [42].

Moreover, the GIP value of the heterogeneous sample is not significantly deviated from the GIP mean value of each range cell. It is easy to lead to inappropriate sample screening and then affect the sample’s selection performance. In the meantime, the GIP method must invert the high-dimension matrix, which requires a large amount of computation. For this reason, many researchers have investigated and improved the GIP method. Such as GIP method was used together with prior knowledge always obtained from actual data [43]. Similarly, this paper aims to solve the shortcomings of the traditional GIP method and improve STAP performance.

As a result, this paper firstly established the echo model and moving target detection model with a side-looking uniform linear array (ULA) for airborne radar. Secondly, this paper analyses the influence of outliers in the training samples on the performance of STAP. Thirdly, constructing the clutter subspace combined with specific distribution in the space-time plane of the clutter spectrum. The clutter subspace is then constructed based on the characteristic [44] that clutter has a specific distribution in the two-dimensional (2-D) space-time plane. Furthermore, there is the construction of the GIP matrix off-line.

Compared with the traditional GIP method, the proposed method constructs the GIP reference matrix off-line based on prior knowledge. In practice, the use of the matrix directly in a practical application does not produce much calculation.

Finally, theoretical analysis and simulation experiments show that the proposed method is easy to build the GIP matrix and requires less computing. Furthermore, it is sensitive to outliers, making it possible to select training samples efficiently.

## 2. The Signal Model

### 2.1. Geometric Model of Space

**Figure 1:**
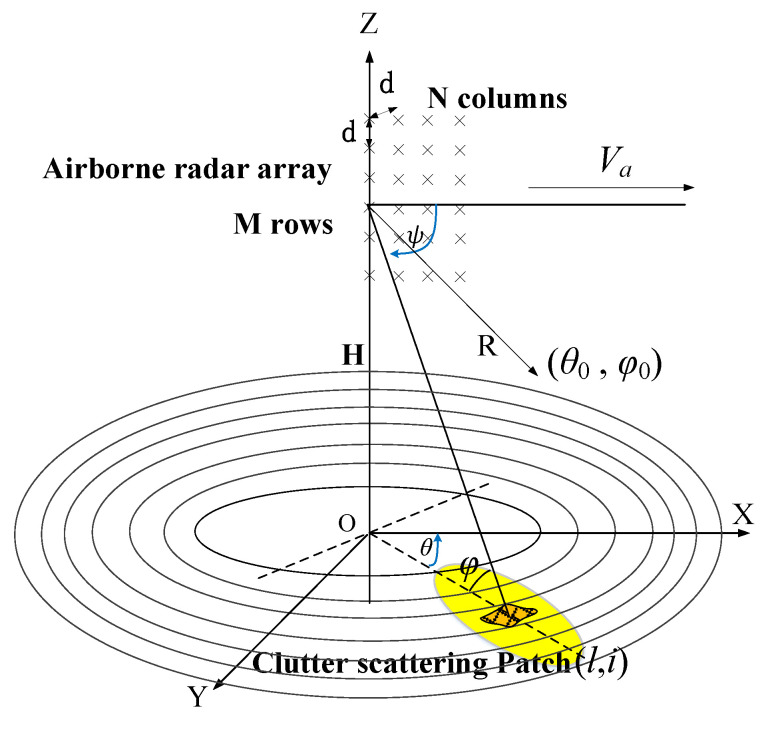
Spatial geometry relation of the airborne multi-channel radar antenna array.

Figure 1 shows the spatial geometric model of the airborne multi-channel radar antenna array. The speed of airborne is *V_a_* and parallel to the direction of the *X*-axis. The wavelength of the radar signal is *λ*. The radar antenna is a planar array of *M* × *N* physical array elements placed uniformly and installed in side-looking. The spacing of array elements is *d* = *λ*/2. The antenna array transmits pulses with a wide aperture. It receives the microwave synthesized echo in rows to obtain a row of the equivalent uniform linear array (ULA) consisting of N equivalent array elements. The primary lobe of the antenna points towards (*θ*_0_, *φ*_0_).

In such a case, the Wald clutter [31] model is adopted to divide the radiated area into grids. This method divides the area covered by a radar beam into *N_c_* equal range cells. It then divides the ring into *N_l_* sufficiently small units called Clutter Scatterer Patch (CSP). The size of each CSP(*l*, *i*) is usually smaller than or equal to the radar’s resolution (*l* = 1, 2, …, *N_c_*; *i* = 1, 2, …, *N_l_*). The antenna gain, the Doppler frequency shift, the slant distance, the direction relative to the radar array, and the Radar Coss-Section (RCS) within each CSP are approximately homogeneous. The RCS of each CSP obeys the Rayleigh distribution. The symbol *θ_l, i_*, and *φ_l_* respectively represent the azimuth-angle and elevation-angle between the CSP(*l*, *i*) and radar array. It ignores the unevenness of the ground and has *φ_l_* = *φ_l, i_*. The symbol *ψ* represents the space cone-angle, and there is an equation cos*ψ_l, i_* = cos*θ_l, i_* ⋅ cos*φ_l_*.

In a Coherent Processing Interval (CPI), *T_r_ = 1/f_r_* is the Pulse Repetition Interval (PRI). The symbol *f_r_* is the pulse repetition frequency (PRF) for radar transmitting signals. Assuming that the symbol *t* is a total time variable, *t_k_* = (*k* − 1)*T_r_* is the azimuth slow-time variable, and $\hat{t}$ = *t* − *t_k_* is the azimuth fast-time variable, (*k* = 1, 2, …, *K*, within *K* is the total number of pulses in a CPI).

The coordinates of the airborne at the time *t*_0_ are (0, 0, H). The coordinates of a point target P within the area covered by the radar beam are (*x*_0_, *y*_0_, H). The symbols *θ_p_* and *φ_p_* respectively represent the azimuth-angle and the elevation-angle between the target P and the radar array. The symbol *ψ_p_* represents the space cone-angle, and there is an equation cos*ψ_p_* = cos*θ_p_* ⋅ cos*φ_p_*.

The symbol *R*_*p,*0_ represents the slant-distance of target P relative to the radar at the time *t*_0_. This document ignores the differences in the space of the array element because the airborne is far from the target relative to the space of the array element.

The target P velocity components in *x*, *y*, and *z*-axes are *v_x_*, *v_y_*, *v_z_*, respectively. The radial velocity of target P relative to the radar is *v_r_*. At the time *t*_0_, the coordinate of *n*-th receiving channels is (*d_n_*, 0, H), where *d_n_* = (*n* − 1)⋅*d*, (*n* = 1, 2, …, *N*).

At the time *t_k_*, the slant-distance *R_n_* (*t_k_*) of target P relative to the *n*-th array element (channel) is:

(1) $$R_{n}\left(t_{k}\right)=\sqrt{{\left[\left(x_{0}+v_{x}t_{k}\right)-\left(d_{n}+V_{a}\cdot t_{k}\right)\right]}^{2}+{\left(y_{0}+v_{y}t_{k}\right)}^{2}+{\left(H-v_{z}t_{k}\right)}^{2}}$$

Based on the geometric model of the space established above, and using Taylor expansion and approximate compensation, the approximate expression of the Equation (1) is:

(2) $$R_{n}\left(t_{k}\right)\approx R_{p,0}+v_{r}t_{k}-V_{a}t_{k}\cdot \cos\theta_{p}\cos\varphi_{p}-d_{n}\cos\theta_{p}\cos\varphi_{p}$$

### 2.2. Echo Signal Model

Assume that *s_T_* ($\hat{t}$, *t_k_*) is the base-band pulse waveform of the radar’s transmitting pulse signal. *f_c_* = *c*/*λ* is the carrier frequency of the radar transmitting signal, where c is the light speed. Within a CPI, the expression of the *k*-th radio-frequency (RF) pulse signal emitted by the radar is:

(3) $$S_{T}\left(\hat{t},t_{k}\right)=s_{T}\left(\hat{t},t_{k}\right)\cdot \exp\left(j2\pi f_{c}\hat{t}\right)$$

It is considering the state of the narrow-band plane wave and ignores the signal envelope walking. Moreover, the expression of the Radio-Frequency (RF) echoes pulse signal *S_R_,_n_* ($\hat{t}$, *t_k_*) of the target P received by *n*-th equivalent array element is:

(4) $$S_{R,n}\left(\hat{t},t_{k}\right)=\xi_{p}\cdot s_{T}\left(\hat{t},t_{k}\right)\exp\left\{j2\pi f_{c}\left[\hat{t}-\tau_{n}\left(t_{k}\right)\right]\right\}$$

The symbol *ξ_p_* represents the echo equivalent amplitude coefficient of the target P. It is composed of RCS, antenna pattern, and system loss. *τ_n_* (*t_k_*) is the bi-directional delay of the echo signal received in the *n*-th equivalent array element. At the time *t_k_*, the expression of the base-band echo signal *s_n_* ($\hat{t}$, *t_k_*) is:

(5) $$s_{n}\left(\hat{t},t_{k}\right)=\xi_{p}\cdot s_{T}\left(\hat{t},t_{k}\right)\exp\left[-j2\pi f_{c}\tau_{n}\left(t_{k}\right)\right]$$

Based on the Wald clutter model [31], in a CPI, the expression of the *k*-th base-band echo pulse of CSP(*l,i*) received by *n*-th equivalent array element is:

(6) $$c_{n,l,i}\left(\hat{t},t_{k}\right)=\xi_{l,i}\cdot s_{T}\left(\hat{t},t_{k}\right)\exp\left[-j2\pi f_{c}\tau_{n,l,i}\left(t_{k}\right)\right]$$

The symbol *ξ_l, i_* represents the echo equivalent amplitude coefficient of CSP(*l,i*), which includes the RCS, antenna pattern, and system loss. *τ_n,l, i_* (*t_k_*) is the bi-directional delay of the echo signal received in the *n*-th equivalent array element.

In the adjacent area around the moving target P in the region covered by the radar beam, the expression of the base-band clutter echo pulse C*_n_* received by the *n*-th equivalent array element at the time *t_k_* is:

(7) $$C_{n}\left(\hat{t},t_{k}\right)=\sum_{l=1}^{N_{c}}\sum_{i=1}^{N_{l}}c_{n,l,i}\left(\hat{t},t_{k}\right)$$

Assume that the radar uses the 1st equivalent array element as both transmit and receive channels simultaneously. Furthermore, the expressions of *τ_n_* (*t_k_*) and *τ_n,l, i_* (*t_k_*) in the Equations (5) and (6) are respectively:

(8) $$\tau_{n}\left(t_{k}\right)=\frac{R_{T}(t_{k})+R_{n}(t_{k})}{c}=\frac{2\left(v_{r}-V_{a}\cos\psi_{a}\right)t_{k}-d_{n}\cos\psi_{a}}{c}+\frac{2R_{p,0}}{c}$$

(9) $$\tau_{n,l,i}\left(t_{k}\right)=\frac{R_{T}(t_{k})+R_{n}(t_{k})}{c}=\frac{-2V_{a}\cos\psi_{l,i}t_{k}-d_{n}\cos\psi_{l,i}}{c}+\frac{2R_{l,i,0}}{c}$$

wherein, *R_T_* (*t_k_*) is the slant-distance of target P relative to the transmitting channel.

From the Equation (8), the separated spatial and time parts are (*d_n_*⋅*cosψ_a_*)*/c* and *2*(*V_a_*⋅*cosψ_a_ − v_r_*)*⋅t_k_/c,* respectively. In addition, the expressions for the spatial angular frequencies *ω_s_* and temporal angular frequencies *ω_t_* belonging to target P are respectively:

(10) $$\omega_{s}=\frac{2\pi }{\lambda }d\cdot \cos\psi_{a}=\frac{2\pi }{\lambda }d\cdot \cos\theta \cos\varphi$$

(11) $$\omega_{t}=\frac{4\pi }{\lambda }\cdot T_{r}\cdot \left(V_{a}\cos\psi_{a}-v_{r}\right)=\frac{4\pi }{\lambda }\cdot T_{r}\cdot \left(V_{a}\cos\theta_{p}\cos\varphi_{p}-v_{r}\right)$$

Moreover, the normalized spatial frequency and temporal frequency (Doppler frequency) are respectively ${\bar{f}}_{s}$ *= ω_s_⋅λ/2π* and ${\bar{f}}_{d}$ = ω*_t_⋅λ/2π*. Meanwhile, there is an equation ${\bar{f}}_{d}$ = *β*⋅${\bar{f}}_{s}$, and *β = 2V/df_r_* is the fold coefficient. Thus, the refined expression for the Equation (5) is:

(12) $$\begin{array}{c} s_{n}\left(\hat{t},t_{k}\right)=\xi_{p}\cdot s_{T}\left(\hat{t},t_{k}\right)\exp\left\{j2\pi f_{c}\left[-\frac{2\left(v_{r}-V_{a}\cos\psi_{a}\right)t_{k}-d_{n}\cos\psi_{a}}{c}\right]\right\}\\ \;\;\;\;\;\;\;\;\;\;\;\;\;\;\;\;\;\;\;\;\;\;\;\;\;\;\;\;\;\;\;\;\;=\xi_{p}\cdot s_{T}\left(\hat{t},t_{k}\right)\exp\left[j\cdot \left(k-1\right)\frac{4\pi }{\lambda }\cdot T_{r}\left(V_{a}\cos\theta_{p}\cos\varphi_{p}-v_{r}\right)\right]\times \\ \;\;\;\;\;\;\;\;\;\;\;\;\;\;\;\;\;\;\;\;\;\;\;\;\;\;\;\;\;\;\;\;\;\;\;\exp\left[j\left(n-1\right)\frac{2\pi }{\lambda }d\cdot \cos\theta_{p}\cos\varphi_{p}\right] \end{array}$$

wherein the symbol *ξ_p_* further includes the constant phase term exp(−4π*R*_*p,*0_*/*λ). Similarly, the refined expression of the Equation (6) is:

(13) $$c_{n,l,i}\left(\hat{t},t_{k}\right)=\xi_{l,i}\cdot s_{T}\left(\hat{t},t_{k}\right)\exp\left[j\left(k-1\right)\frac{4\pi }{\lambda }V_{a}\cdot T_{r}\cos\theta_{i}\cdot \cos\varphi_{l}\right]\exp\left[j\left(n-1\right)\frac{2\pi }{\lambda }d\cos\theta_{i}\cdot \cos\varphi_{l}\right]$$

The symbol *ξ_l, i_* additionally includes the constant phase term exp(−4π*R*_*l,i,*0_*/*λ).

### 2.3. Space-Time Steering Vector and Space-Time Clutter Spectrum Model

The temporal domain direction vector of the target P is:

(14) $$\begin{array}{c} S_{t}{\left(\omega_{t}\right)}_{K\times 1}={\left\{1,\exp\left[j\omega_{t}\right],\cdots ,\exp\left[j\left(K-1\right)\omega_{t}\right]\right\}}^{T}\\ \;\;\;\;\;\;\;\;\;\;\;\;\;\;\;\;\;\;\;\;\;\;\;\;\;\;\;\;\;\;\;\;\;\;\;\;\;\;=\{\begin{array}{ccc} 1, & \exp\left[j\frac{4\pi }{\lambda }\cdot T_{r}\cdot \left(V_{a}\cos\theta_{p}\cos\varphi_{p}-v_{r}\right)\right], & \cdots , \end{array}\\ \;\;\;\;\;\;\;\;\;\;\;\;\;\;\;\;\;\;\;\;\;\;\;\;\;\;\;\;\;\;\;\;\;\;\;\;\;\;\;\;\;\;\;\;\;{\begin{array}{cc} \cdots , & \exp\left[j\left(K-1\right)\frac{4\pi }{\lambda }\cdot T_{r}\cdot \left(V_{a}\cos\theta_{p}\cos\varphi_{p}-v_{r}\right)\right] \end{array}\}}^{T} \end{array}$$

Meanwhile, the spacial steering vectors of target P is:

(15) $$\begin{array}{c} S_{s}{\left(\omega_{s}\right)}_{N\times 1}={\left\{1,\exp\left[j\omega_{s}\right],\cdots ,\exp\left[j\left(N-1\right)\omega_{s}\right]\right\}}^{T}\\ \;\;\;\;\;\;\;\;\;\;\;\;\;\;\;\;\;\;={\left\{1,\exp\left[j\frac{2\pi }{\lambda }d\cdot \cos\theta_{p}\cos\varphi_{p}\right],\cdots ,\exp\left[j\left(N-1\right)\frac{2\pi }{\lambda }d\cdot \cos\theta_{p}\cos\varphi_{p}\right]\right\}}^{T} \end{array}$$

As well, the space-time steering vector of target P is:

(16) $$\begin{array}{c} S_{P}=S_{t}{\left(\omega_{t}\right)}_{K\times 1}⊗S_{s}{\left(\omega_{s}\right)}_{N\times 1}\\ ={\left[\begin{array}{c} 1\\ \vdots \\ \exp\left[j\left(N-1\right)\omega_{s}\right]\\ \exp\left[j\frac{4\pi }{\lambda }\cdot T_{r}\left(V_{a}\cos\theta_{p}\cos\varphi_{p}-v_{r}\right)\right]\\ \vdots \\ \exp\left[j\left(N-1\right)\frac{2\pi }{\lambda }d\cos\theta_{p}\cos\varphi_{p}\right]\exp\left[j\frac{4\pi }{\lambda }\cdot T_{r}\left(V_{a}\cos\theta_{p}\cos\varphi_{p}-v_{r}\right)\right]\\ \vdots \\ \exp\left[j\left(K-1\right)\omega_{t}\right]\\ \vdots \\ \exp\left[j\left(N-1\right)\frac{2\pi }{\lambda }d\cos\theta_{p}\cos\varphi_{p}\right]\exp\left[j\left(K-1\right)\frac{4\pi }{\lambda }\cdot T_{r}\left(V_{a}\cos\theta_{p}\cos\varphi_{p}-v_{r}\right)\right] \end{array}\right]}_{\mathrm{NK}\times 1} \end{array}$$

The vector expression of the under-tested range samples in a CPI is an NK × 1 dimension vector:

(17) $$x={\left[x_{s}^{T}\left(1\right),x_{s}^{T}\left(2\right),\ldots ,x_{s}^{T}\left(K\right)\right]}^{T}$$

wherein, **x_s_**(*k*) = [*x*_1_ ($\hat{t}$, *t_k_*), *x*_2_ ($\hat{t}$, *t_k_*), …, *x_n_* ($\hat{t}$, *t_k_*)]^T^, and *x_n_* ($\hat{t}$, *t_k_*) represents the *k*-th base-band echo pulse data obtained by the *n*-th equivalent array element (*n* = 1, …, *N, k =* 1, 2, …, *K*).

The received signal **x** generally consists of the target echo signal **s**, the clutter signal **c**, and the noise signal **n**, that is, **x** = **s** + **c** + **n**. Depending on the definition of the Equation (17), their expressions are respectively:

(18) $$s={\left[s_{1}\left(\hat{t},t_{1}\right),\ldots ,s_{N}\left(\hat{t},t_{1}\right),\ldots ,s_{1}\left(\hat{t},t_{K}\right),\ldots ,s_{N}\left(\hat{t},t_{K}\right)\right]}^{T}$$

(19) $$c={\left[C_{1}\left(\hat{t},t_{1}\right),\ldots ,C_{N}\left(\hat{t},t_{1}\right),\ldots ,C_{1}\left(\hat{t},t_{K}\right),\ldots ,C_{N}\left(\hat{t},t_{K}\right)\right]}^{T}$$

(20) $$n={\left[n_{1}\left(\hat{t},t_{1}\right),\ldots ,n_{N}\left(\hat{t},t_{1}\right),\ldots ,n_{1}\left(\hat{t},t_{K}\right),\ldots ,n_{N}\left(\hat{t},t_{K}\right)\right]}^{T}$$

wherein, n*_n_* ($\hat{t}$, *t_k_*) represents the noise component in the *k*-th base-band echo pulse data obtained by the *n*-th equivalent array element. Supposing that the noise obeys a zero-mean Gaussian distribution and the variance is *σ*^2^. The space-time sample covariance matrix (ST-SCM) for the under-tested range sample is:

(21) $$R_{cn}=E\left[\left(c+n\right){\left(c+n\right)}^{H}\right]=R_{c}+\sigma^{2}I$$

The symbol **R_c_** is the space-time clutter covariance matrix (ST-CCM). The symbol **I** is the identity matrix of the dimensions N × K.

### 2.4. Optimum Space-Time Processing

**Figure 2:**
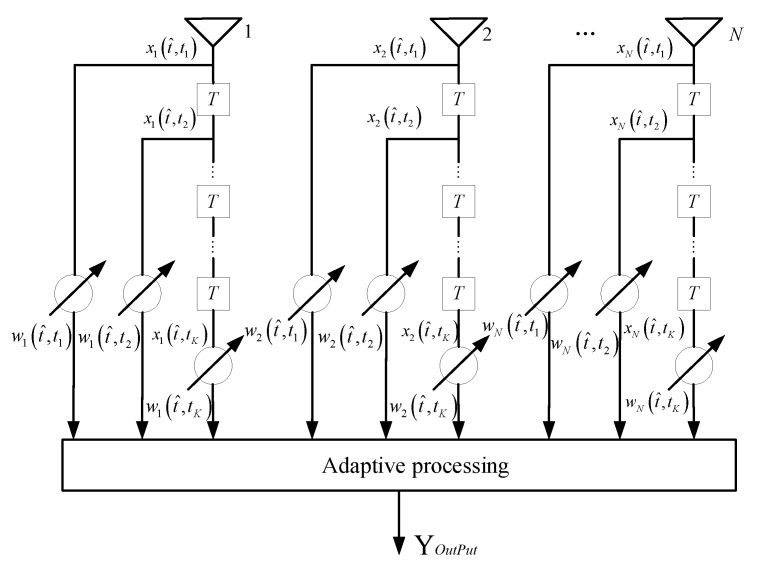
Structure diagram of Space-time Adaptive Processing (STAP).

Figure 2 illustrates the structure of STAP structure. According to Figure 2, the inner product of the optimum weight vector **w***_opt_* and the under-tested range sample is the expression of the **Y***_opt_* of the optimum STAP output:

(22) $$Y_{out}=w_{opt}^{H}x$$

wherein, the expression of **w***_opt_*:

(23) $$w_{opt}=\mu R_{cn}^{-1}S_{P}$$

Corresponding to Figure 2, the other expression of the **w***_opt_* is:

(24) $$w_{opt}={\left[\begin{array}{llllllllll} w_{1}\left(\hat{t},t_{k}\right) & \cdots & w_{N}\left(\hat{t},t_{1}\right) & w_{1}\left(\hat{t},t_{2}\right) & \cdots & w_{N}\left(\hat{t},t_{2}\right) & \cdots & w_{1}\left(\hat{t},t_{k}\right) & \cdots & w_{N}\left(\hat{t},t_{K}\right) \end{array}\right]}^{T}$$

In the Equation (23), the **R***_cn_* can not be directly available. The radar detection system can obtain L vectors data following the IID condition as the training samples. That is the sample data in the adjacent range cells around the target P. Therefore, the expression of the maximum likelihood estimation (MLE) of **R***_cn_*:

(25) $${\overset{⌢}{R}}_{cn}=E\left[xx^{H}\right]\approx \left(1/L\right)\sum_{l=1}^{L}x_{l}x_{l}^{H}$$

The expression of filter weight vector estimation through the MLE of **R***_cn_*:

(26) $${\overset{⌢}{w}}_{opt}=\mu {\overset{⌢}{R}}_{cn}^{-1}S_{P}$$

wherein the expression of **μ** is

(27) $$\mu =1/\left(S_{P}^{H}R_{c}^{-1}S_{P}\right)$$

**Figure 3:**
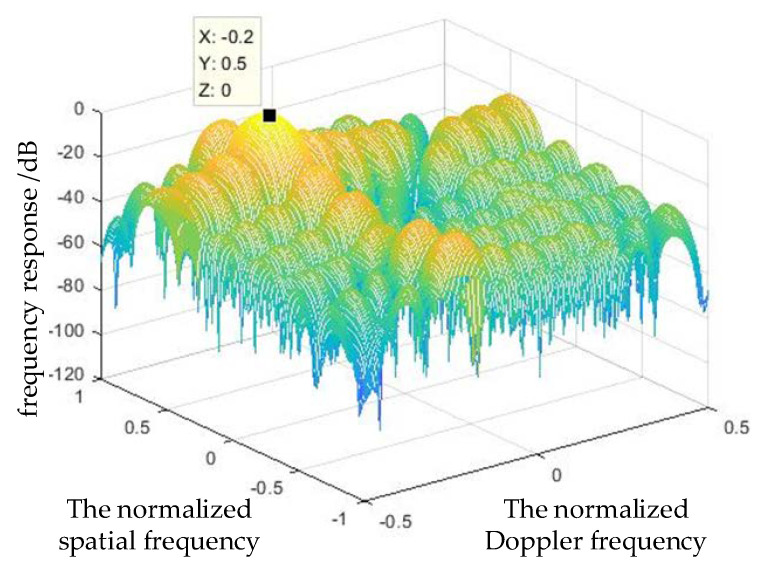
The 2−D frequency response of the optimum STAP.

The function of the adaptive model formed by the optimum STAP is **F = $w_{\mathrm{opt}}^{H}$S**. Therefore, it is possible to obtain the adaptive model(2-D frequency response) by calculating the optimum weight vector. Figure 3 shows that the adaptive model forms notch distributed through the clutter, effectively filtering out the clutter in the ideal conditions.

Based on the Equations (21), (23), (25)–(27), one can obtain the output SCNR of the optimum weight vector and the estimation of the weight vector, respectively:

(28) $$\mathrm{SCNR}\_w_{opt}={\left|\xi_{p}\right|}^{2}S_{P}^{H}R_{cn}^{-1}S_{P}$$

(29) $$\mathrm{SCNR}\_{\overset{⌢}{w}}_{opt}={\left|\xi_{p}\right|}^{2}\frac{{\left(S_{P}^{H}{\overset{⌢}{R}}_{cn}^{-1}S_{P}\right)}^{2}}{S_{P}^{H}{\overset{⌢}{R}}_{cn}^{-1}R_{cn}{\overset{⌢}{R}}_{cn}^{-1}S_{P}}$$

According to the Equations (28) and (29), the expression of the output SCNR loss is:

(30) $$\rho_{{\overset{⌢}{w}}_{opt}}=\frac{\mathrm{SCNR}\_{\overset{⌢}{w}}_{opt}}{\mathrm{SCNR}\_w_{opt}}=\frac{{\left(S_{P}^{H}{\hat{R}}_{cn}^{-1}S_{P}\right)}^{2}}{S_{P}^{H}{\hat{R}}_{cn}^{-1}R_{cn}{\hat{R}}_{cn}^{-1}S_{P}}\cdot \frac{1}{S^{H}R_{cn}^{-1}S_{P}}$$

## 3. Influence of Outfilers in Training Samples on STAP Performance

When there are outliers, it needs to obtain the ST-CCM expression of the under-tested range samples. Assuming that there is no correlation between the outliers and the clutter plus noise data, using the Equation (25), the ST-CCM expression is:

(31) $$R_{outlier}={\overset{⌢}{R}}_{cn}+\sum_{j=1}^{N_{j}}\xi_{j}S_{I_{j}}S_{I_{j}}^{H}$$

where *ε_j_* and **S***_Ij_* are the complex-amplitude and space-time steering vector corresponding to the *j*-th outlier, respectively. *N_j_* is the total number of outliers. For the convenience of analysis, assuming there is only one outlier. The expression of the eigendecomposition of **R***_outlier_* is:

(32) $$R_{outlier}=\sum_{i=1}^{NK}\lambda_{i}u_{i}u_{i}^{H}$$

where, *λ*_1_ ≥ ⋯ ≥ *λ_Rank_*_(_**_Ȓ_***_cn_*_) + 1_ ≥ *λ_Rank_*_(_**_Ȓ_***_cn_*_) + 2_ = ⋯ = *λ_NK_* = *σ*^2^ is the eigenvalues of **R***_outlier_*, **u***_i_* is the eigenvector corresponding *i*-th outlier eigenvalue. Depending on the relationship between signal subspace and noise subspace [45,46], an equation exists:

(33) $$\sum_{i=Rank({\hat{R}}_{cn})+2}^{NK}\lambda_{i}u_{i}u_{i}^{H}=I-\sum_{i=1}^{Rank({\hat{R}}_{cn})+1}\lambda_{i}u_{i}u_{i}^{H}$$

Using the Equation (33), we can obtain further:

(34) $$R_{outlier}^{-1}=\sum_{i=1}^{Rank({\hat{R}}_{cn})+1}\frac{1}{\lambda_{i}}u_{i}u_{i}^{H}+\sum_{i=Rank({\hat{R}}_{cn})+1}^{NK}\frac{1}{\sigma^{2}}u_{i}u_{i}^{H}=\frac{1}{\sigma^{2}}\left(I-\sum_{i=1}^{Rank({\hat{R}}_{cn})+1}\frac{\lambda_{i}-\sigma^{2}}{\lambda_{i}}u_{i}u_{i}^{H}\right)$$

Without loss of generality, assuming a higher Clutter to Noise Ratio (CNR), in other words, there is *λ_i_* >> *σ*^2^, *i* = 1, …, *r*. Then, the other expression of the Equation (34) is:

(35) $$R_{outlier}^{-1}\approx \frac{1}{\sigma^{2}}\left(I-\sum_{i=1}^{Rank({\hat{R}}_{cn})+1}u_{i}u_{i}^{H}\right)=\frac{1}{\sigma^{2}}\sum_{i=Rank({\hat{R}}_{cn})+2}^{NK}u_{i}u_{i}^{H}$$

The function of the Equation (35) is to whiten the input data. In other words, it can project the input data into the noise subspace. According to the orthogonality between the signal subspace and the noise subspace, the filter’s weight vector will produce notches at the outlier to suppress them. However, suppose the outlier in the training samples is in the signal subspace. In such a case, the filter will also suppress the under-detected targets.

Therefore, suppose the outlier has strong coherence with the under-tested targets. In other words, the 2-D space-time position information (including direction and Doppler) is very close for the outlier and under-detected targets in the training samples. At this time, the filter weight vector will produce the deep notch to cancel the under-detected targets and outlier simultaneously, which will reduce the target detection performance of STAP.

Considering the influence on the SATP output by the outlier in the training samples, and according to the Equation (30), the expression of the output SCNR loss is:

(36) $$\rho_{outlier}=\frac{{\left(S_{P}^{H}R_{outlier}^{-1}S_{P}\right)}^{2}}{S_{P}^{H}R_{outlier}^{-1}R_{cn}R_{outlier}^{-1}S_{P}}\cdot \frac{1}{S_{P}^{H}R_{cn}^{-1}S_{P}}$$

**Figure 4:**
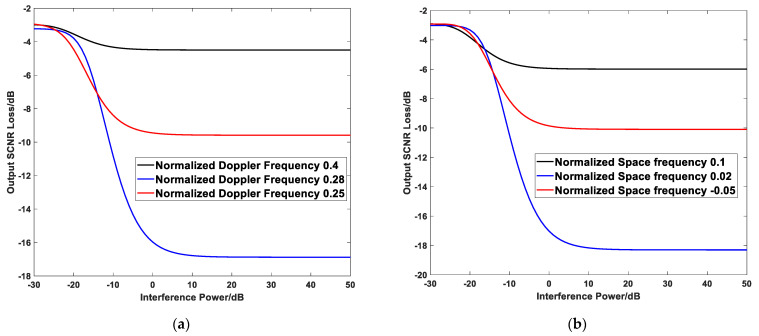
Effects of the outlier on output SCNR losses in training samples. They should be listed as: (**a**) Output SCNR losses at different normalized Doppler frequencies; (**b**) Output SCNR losses at different normalized space frequencies.

Figure 4a,b present the output SCNR loss simulation results varying with outlier power under different normalized Doppler frequencies and normalized spatial frequencies of the outlier. Table 1 provides the radar system simulation parameters.

The simulation results in Figure 4 show that the wider the 2-D space-time position between the outlier and the under-detected targets. In other words, the weaker the correlation between them, the smaller the output SCNR loss. By comparison, the closer the 2-D space-time position between the outlier and the under-detected targets are, in other words, the stronger the correlation between them, the greater the output SCNR loss.

Moreover, with the increase of outlier power (as the interference power), the loss of output SCNR remains stable after increasing to a certain extent. The reason is that the interference component’s character in training samples transforms from non-significant to significant gradually. In other words, the eigenvalues of it transform from small to large.

It has happened that the transforming process canceled the target gradually. Furthermore, when the interference power increases to a certain extent, the end canceled the target ultimately. At this point, the output SCNR loss remains stable.

## 4. A Sample Selection Method Based on Clutter Subspace

According to the analysis of Section 3, the outlier will lead to the cancellation of target signals, resulting in a significant loss of output SCNR and ultimately leading to a decline in the radar detection performance. To more accurately estimate the ST-CCM of the under-tested rang sample data, it is inevitable to select appropriate training samples.

Melvin et al. proposed a method for selecting training samples based on the Generalized Inner Product (GIP). It is known as the traditional GIP method. This method sets the GIP test statistics and then tests each training sample separately. Comparing the GIP value of the training samples containing the outlier with not, they are significantly different. The traditional GIP method can effectively find the training samples with outliers to eliminate them before estimating the ST-CCM.

However, the performance of the traditional GIP method decreases obviously in the heterogeneous clutter environment. Besides, its computation is extensive. For this reason, this section starts with the basic principle of the traditional GIP method. It then proposes a method of sample selection based on clutter subspace.

### 4.1. Traditional GIP Method

The GIP method proposed by Melvin et al. is one of the most common sample selection methods. Its basic principle is selecting and removing the training samples whose statistical characteristics are different from those of the under-tested range sample data by GIP value. Firstly, the following gives the definition:

**Definition** **1.** *Assuming that***X**_*i*_*and***X**_*j*_*have the same dimension,***R**_*i*_*and***R**_*j*_*are their self-correlation matrices, respectively. If the following formula is true*:

(37) $$R_{i}R_{j}^{-1}\approx I$$

Then, the statistical distribution of the vectors **X***_i_* and **X***_j_* are identical or approximately identical. In other words, the statistical characteristic between them is homogeneous. Otherwise, **X***_i_* and **X***_j_* are singular or homogeneous. For the *i*-th training sample, the expression of its GIP value is:

(38) $$\eta_{GIP,i}=x_{i}^{H}R_{cn}^{-1}x_{i}={\left|R_{cn}^{-1/2}x_{i}\right|}^{2}$$

According to the Equation (38), the GIP value’s physical sense is the inner product of the vector of training samples whitened by matrix **$R_{cn}^{-1/2}$**. We call the matrix **R***_cn_* to compute the GIP value as the GIP reference matrix. Assuming that the ST-CCM of the *i*-th training sample is **R***_cn,i_*, we can furtherly obtain the expression of the Equation (38) as follows:

(39) $$E\left[\eta_{GIP,i}\right]=E\left[x_{i}^{H}R_{cn}^{-1}x_{i}\right]=trace\left\{E\left[R_{cn}^{-1}x_{i}x_{i}^{H}\right]\right\}=trace\left\{R_{cn}^{-1}R_{cn,i}\right\}$$

According to the Equation (39) and Definition 1, if the statistical characteristics of *i*-th training sample and the under-tested rang sample data are homogeneous, then there is an equation:

(40) $$E\left[\eta_{GIP,i}\right]=trace\left\{R_{cn}^{-1}R_{cn,i}\right\}\approx trace\left\{I\right\}=NK$$

On the contrary, the value of (39) will deviate from *NK*. The traditional GIP method judges each training sample’s homogeneous degree by detecting the offset degree of the GIP value of the training samples relative to the *NK*.

In practice, the GIP matrix is unknown because we cannot obtain the accurate ST-CCM. Therefore, the traditional GIP method usually uses the training samples on both sides of the under-tested sample to estimate the GIP matrix.

However, in the heterogeneous clutter environment, the GIP reference matrix obtained is seriously mismatched. The reason is that each training sample’s heterogeneity is variable, and there is the shielding effect of the potential outlier. Meanwhile, the offset degree is not apparent between the GIP value of the heterogeneous training samples and the GIP mean value of each under-tested range sample. Moreover, it quickly leads that the training samples selected are unsuitable and then reduces the selection performance of the traditional GIP method for the heterogeneous training samples.

### 4.2. Clutter Subspace Feature-Assisted Sample Selection Method

**Figure 5:**
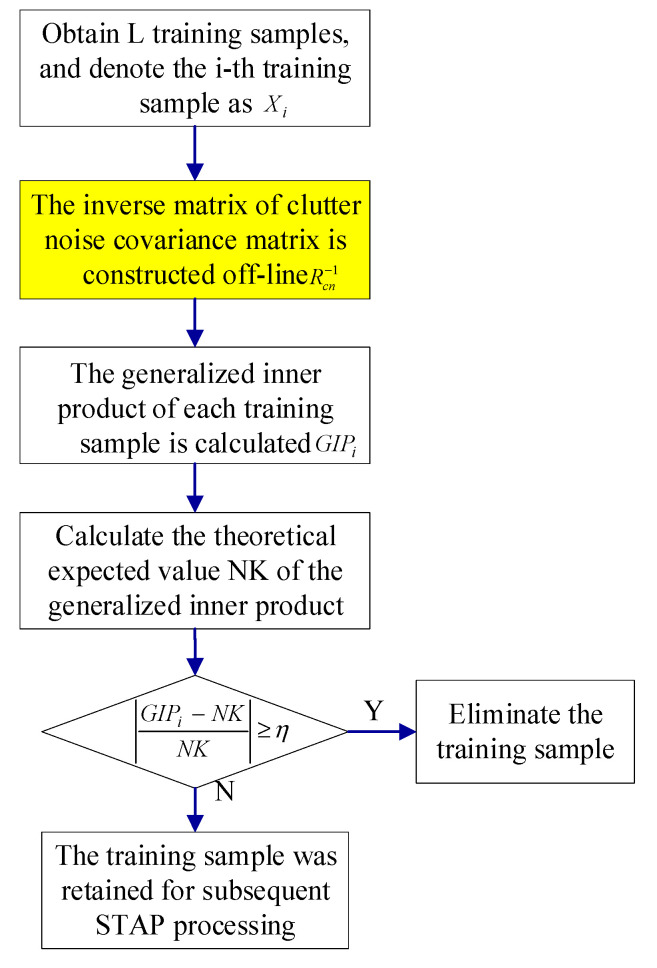
The flow chart for the clutter subspace characteristics-aided sample selection method.

The traditional GIP method has significant limitations because the training samples’ heterogeneity and outlier parameters can affect its performance efficiently. Simultaneously, it needs to invert the matrix, so the amount of calculation is large. Considering that the airborne radar clutter with the side-looking ULA has a specific distribution in the 2-D space-time plane, this paper proposes a sample selection method based on the clutter subspace. Figure 5 illustrates the flowchart for the method proposed in this paper.

The Equations (39) and (40) and Figure 5 show that the key to the method proposed in this article is to estimate the GIP reference matrix **R***_cn_* or its inverse matrix $R_{cn}^{-1}$. The method proposed in this paper adopts the off-line construction of the matrix $R_{cn}^{-1}$ to replace the traditional GIP method used to estimate the matrix **R***_cn_* based on the under-tested sample, as the yellow box in the Figure 5.

According to the Equation (34), the clutter subspace or noise subspace can construct the expression of $R_{cn}^{-1}$:

(41) $$R_{cn}^{-1}\approx \frac{1}{\sigma^{2}}\left(I-U_{c}U_{c}^{H}\right)=\frac{1}{\sigma^{2}}U_{n}U_{n}^{H}$$

wherein **U***_c_* and **U***_n_* are the actual clutter and the noise subspace of the under-tested range samples, respectively. According to the Equation (41), if constructing the clutter or noise subspace of the GIP reference matrix accurately, it is easy to obtain $R_{cn}^{-1}$. For clutter received by the radar, the clutter subspace has the following properties:

**Property** **1.** Considering without the non-ideal factors, the subspace of the clutter signal received by the radar is independent of the RCS of the CSPs. It only depends on the space-time steering vector of the CSP, that is:

(42) $$Span(R_{c})=Span(S_{c})$$

wherein, **S**_c_ is a matrix composed of the space-time steering vectors corresponding to each CSP.

**Proof** **of** **Property** **1.** The ST-CCM of clutter is equivalent to:

(43) $$R_{c}=S_{c}\Lambda S_{c}^{H}$$

wherein, **Λ** is a diagonal matrix composed of the square of each CSP’s complex amplitude. According to the subspace span theorem [39], there is an expression:

(44) $$Span(R_{c})=Span(S_{c})$$

The expression of the space-time steering vector of a single CSP at (*f_s_*, ${\bar{f}}_{d}$) is [38]:

(45) $$S({\bar{f}}_{s},{\bar{f}}_{d})={\left[\begin{array}{c} 1\\ e^{j2\pi {\bar{f}}_{s}}\\ \vdots \\ e^{j2\pi (N-1){\bar{f}}_{s}}\\ e^{j2\pi {\bar{f}}_{d}}\\ e^{j2\pi ({\bar{f}}_{d}+{\bar{f}}_{s})}\\ \vdots \\ e^{j2\pi ({\bar{f}}_{d}+(N-1){\bar{f}}_{s})}\\ e^{j2\pi 2{\bar{f}}_{d}}\\ e^{j2\pi (2{\bar{f}}_{d}+{\bar{f}}_{s})}\\ \vdots \\ e^{j2\pi ((K-1){\bar{f}}_{d}+(N-1){\bar{f}}_{s})} \end{array}\right]}_{\mathrm{NK}\times 1}$$

For a side-looking airborne radar with the ULA, the space-time clutter spectrum is linear, and responds to: ${\bar{f}}_{d}$ = *β ∙* ${\bar{f}}_{s}$. Assuming that *β* is an integer, the further expression of the Equation (45) is:

(46) $$S({\bar{f}}_{s},{\bar{f}}_{d})={\left[\begin{array}{c} 1\\ e^{j2\pi {\bar{f}}_{s}}\\ \vdots \\ e^{j2\pi (N-1){\bar{f}}_{s}}\\ e^{j2\pi \beta {\bar{f}}_{s}}\\ e^{j2\pi (\beta +1){\bar{f}}_{s}}\\ \vdots \\ e^{j2\pi (\beta +(N-1)){\bar{f}}_{s}}\\ e^{j2\pi 2\beta {\bar{f}}_{s}}\\ e^{j2\pi (2\beta +1){\bar{f}}_{s}}\\ \vdots \\ e^{j2\pi (\beta (K-1)+(N-1)){\bar{f}}_{s}} \end{array}\right]}_{\mathrm{NK}\times 1}=E\cdot {\left[\begin{array}{c} 1\\ e^{j2\pi {\bar{f}}_{s}}\\ \vdots \\ e^{j2\pi (\beta (K-1)+(N-1)){\bar{f}}_{s}} \end{array}\right]}_{\left[N+\beta (K-1)\right]\times 1}$$

In the Equation (46), **E** is an *NK* × [*N* + *β*(*K*−1)] dimensional matrix, whose elements in the *i*-th row and *j*-th column are:

(47) $$e_{ij}=\left\{ \begin{array}{l} \begin{array}{cccc} 1 & i=(k-1)N+n & and & j=n+\beta (k-1) \end{array}\\ \begin{array}{cc} 0 & others \end{array} \end{array}\right.$$

wherein, *n* = 1, …, *N*; *k* = 1, …, *K*. The element in the (*k* − 1)*N* + *n* row and the *n* + *β*(*k* − 1) column is 1. The other elements are 0, so the **E** is an orthonormal column matrix. Combining with Property 1, the linear combination of *N* + *β*(*k* − 1) orthogonal column vectors of **E** can construct the space-time steering vector constructed by **S***_c_* in the clutter subspace. Therefore, the column space of matrix **E** is the clutter subspace. To construct the orthonormal basis of **E**, we normalize the column vectors of **E** to obtain matrix **E***_c_*, and then there is an equation:

(48) $$Span(R_{c})=Span(U_{c})=Span(E)=Span(E_{c})$$

According to the Equation (48), the column spaces of the matrices **U***_c_* and **E***_c_* belong to the same clutter subspace. Depending on the properties of the subspace, the linear combination with the column vectors of **E**_*c*_ can construct the column vectors of **U***_c_*, as follows:

(49) $$U_{c}=E_{c}Q$$

Based on the orthogonality of the column vectors of the **U***_c_* and **E***_c_*, there is:

(50) $$U_{c}^{H}U_{c}=Q^{H}E_{c}^{H}E_{c}Q=Q^{H}Q=I$$

The Q is an orthogonal matrix, so:

(51) $$U_{c}U_{c}^{H}=E_{c}QQ^{H}E_{c}^{H}=E_{c}E_{c}^{H}$$

By substituting the Equation (51) into (41), the GIP reference matrix is as follows:

(52) $$R_{cn}^{-1}\approx \frac{1}{\sigma^{2}}\left(I-U_{c}U_{c}^{H}\right)=\frac{1}{\sigma^{2}}\left(I-E_{c}E_{c}^{H}\right)$$

By substituting the Equation (52) into (38), the GIP value is, as follows:

(53) $$\eta_{\mathrm{proposed},i}=x_{i}^{H}R_{cn}^{-1}x_{i}=\frac{1}{\sigma^{2}}x_{i}^{H}\left(I-E_{c}E_{c}^{H}\right)x_{i}$$

According to the Equations (40) and (53), when there is a relatively close homogeneous degree between the *i*-th training sample and the under-tested rang samples (e.g., there is no outlier), the GIP value of the training sample will be close to *NK*. Otherwise, the GIP value will deviate from the NK value, and there should be an elimination of the training samples.

In the process of constructing the GIP reference matrix, we can obtain the noise power in advance. Simultaneously, the clutter subspace E_*c*_ elements are independent of the pitch angle, which applies to all under-tested range samples. Moreover, according to the specific distribution of clutter in 2-D space-time plane and radar system parameters based on the side-looking with the ULA, an E_*c*_ can be constructed off-line.

These methods reduce the amount of calculation and avoid an inaccurate estimation of the GIP reference matrix due to outlier existence. The proposed method is more sensitive to the outlier, so heterogeneous training samples’ elimination performance is enhanced.

## 5. Simulation Experiment and Analysis

In this section, there is a simulation experiment to perform the proposed method. Assuming that the aircraft’s flight altitude is 9000 m; the flight speed is 50 m/s; the transmission signal wavelength is 0.667 m, and the PRF is 300 Hz. The radar antenna is side-looking with ULA. Moreover, each array element is equal to half of the wavelength; the array element number is 8; the number of pulses received in a CPI is 8. The number of training samples used in the simulation experiment is 141. There is the target located in the 71st range cell. The normalized Doppler frequency corresponding to the target is 0.25; the airspace frequency is 0; the SNR is 20 dB.

**Figure 6:**
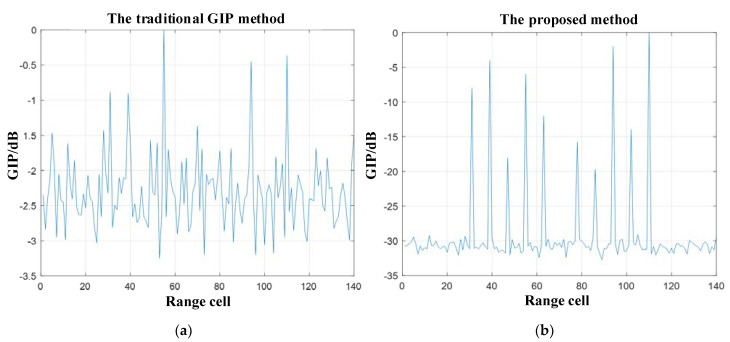
The simulation results of GIP value and output SCNR, they should be listed as: (**a**) GIP value simulation results of the traditional GIP method; (**b**) Simulation results of the GIP value of the proposed method in this paper.

Furthermore, assuming the CNR is 40 dB, five outliers as interferences are added randomly to both sides of the under-tested range cell. The normalized Doppler frequency of the interference is equivalent to the target. In addition, the direction of the interferences is random, but within the main lobe; Table 2 shows the range cell and the interference to noise ratio (INR) of the interference:

Figure 6a represents the GIP value simulation results of the traditional method, and Figure 6b represents the GIP value simulation results of the proposed method. The simulation results in Figure 6 show that the traditional GIP method is not robust for detecting interferences. The reason is that there are no apparent offsets comparing the GIP values of the range cells containing most of the interferences with not. However, the GIP values of range cells containing a small number of solid interferences have a larger offset than the others. Therefore, the selection of detection threshold should not be small. Otherwise, it will lead to the elimination of more homogeneous samples and reduce the detection performance.

By comparison, the method proposed in this paper has a good detecting effect for the training samples. The GIP values of all the range cells containing the interferences have a large offset relative to other range cells. They reflect the weak and robust information about the interferences. Consequently, the proposed method’s detection threshold set is large, making it difficult to eliminate the homogeneous samples.

**Figure 7:**
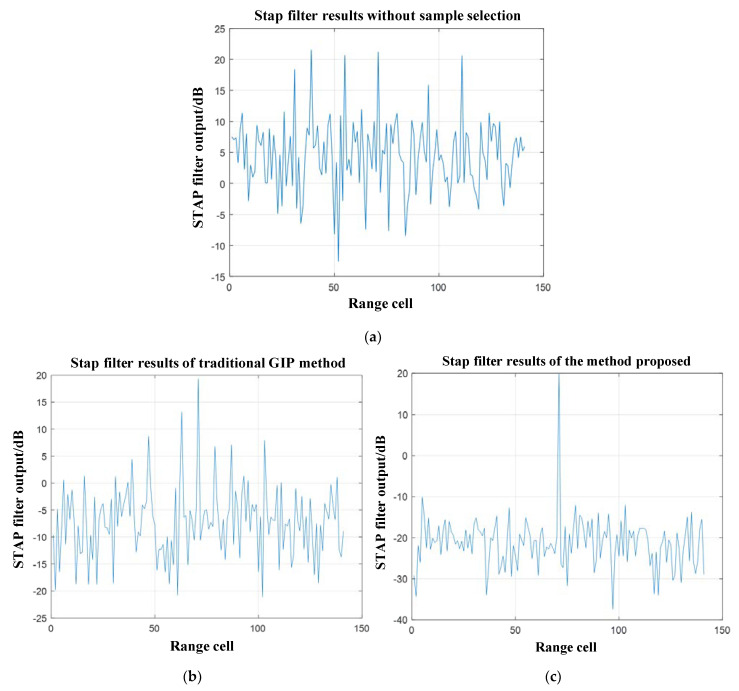
The simulation results of the STAP filter, they should be listed as: (**a**) without sample selection; (**b**) with the traditional GIP method; (**c**) with the method proposed in this paper.

Furtherly, Figure 7a–c respectively show the clutter suppression results of STAP with the methods: (a) without sample selection, (b) with the traditional GIP method, and (c) with the method proposed in this paper.

Figure 7a shows that the method without sample selection cannot detect any targets. Figure 7b shows that the position corresponding to the maximum output power value of the STAP filter obtained by the traditional GIP method is the same as that of the target. However, for most of the range cells, the output power corresponding STAP filter is also very high. It does not detect and eliminate the interference effectively. Moreover, the output power of STAP at the target position generates an SNR loss (relative to the set SNR = 20 dB). Therefore, it cannot guarantee the performance of target detection.

By comparison, in Figure 7c, the STAP filter obtained the maximum output power located in the range cell containing the target by the method proposed in this paper. In the meanwhile, there is almost no SNR loss. Furthermore, the STAP filter suppresses output power corresponding to other range cells obviously. It demonstrates that the method proposed in this paper can effectively detect and eliminate interference to ensure STAP performance.

**Figure 8:**
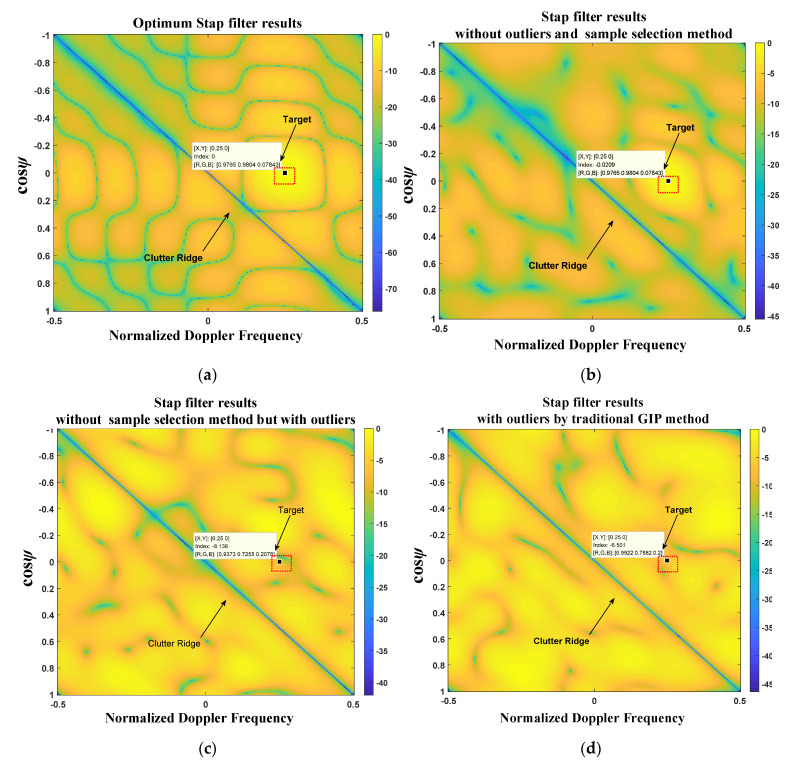

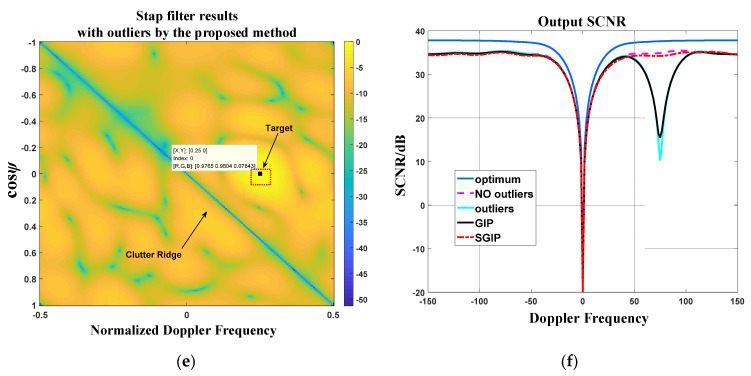
Simulation results of 2D space-time frequency response, they should be listed as: (**a**) Optimum STAP filter results (with actual ST−CCM); (**b**) STAP filter results without outliers and any sample selection method; (**c**) STAP filter results without any sample selection method but with outliers; (**d**) STAP filter results with outliers by traditional GIP method; (**e**) STAP filter results with outliers by the proposed method in this paper; (**f**) Comparison results of SCNR output by STAP filters with different methods.

Figure 8a–e presents the 2-D simulation results of space-time frequency response by five methods. These more intuitively illustrate the advantages of the method proposed in this paper. Figure 8f further presents the simulation quantization comparison results of SCNR output by STAP filters with different methods.

Figure 8a shows the theoretically optimum STAP filter results. The target response is 0 dB. Moreover, there is a deep notch in the clutter;

Figure 8b shows the STAP filter results with the sample matrix inverse (SMI) method when there is no outlier. The target response is 0 dB approximately. Moreover, there is a deep notch in the clutter, but not as deep as the Figure 8a;

Figure 8c shows the STAP filter results without any sample selection method when there are outliers. The target has a prominent cancellation. The response is less than 0 dB;

Figure 8d shows the STAP filter results using the traditional GIP method when there are outliers. The target has a slight cancellation. The response is less than 0 dB; Figure 8e shows the STAP filter results according to the proposed method in this article when there are outliers. The target response is 0 dB. Additionally, there is a deep notch in the clutter, almost the same as in Figure 8b.

The proposed method does not produce cancellation to the target. Furthermore, it has the same performance as the case without outliers. It only loses 3 dB compared with the theoretical optimum STAP filter results, which conforms to the theoretical analysis. These indicate that the method proposed has a good detection performance.

## 6. Conclusions

The heterogeneous clutter environment faced by airborne radar may make outliers exist in the training samples. Therefore, the statistical characteristics between the training samples and the under-tested rang samples no longer satisfy the IID conditions, making the calculation inaccurate of the weight vector. Moreover, it causes the cancellation of the under-detected targets and deduces the detection performance of the targets. Therefore, it is necessary to eliminate the outliers’ training samples before estimating the space-time correlation matrix.

This paper analyzed the effects of the outlier on STAP performance and then introduced the traditional GIP method. This paper proposed a sample selection method based on clutter subspace, considering the specific distribution on the 2-D space-time plane of clutter received by the airborne radar with the side-looking ULA. The method proposed in this paper is to solve significant computation amount problems and be affected by outlier parameters when constructing the GIP reference matrix for the traditional GIP method.

This method can construct the GIP reference matrix off-line and has the characteristics of small computation. Moreover, it is sensitive to the outlier and has good performance in sample selection. Theoretical analysis and simulation experiments support the effectiveness of the proposed algorithm.

## Figures and Tables

**Table 1:** Radar system simulation parameters.

Parameter	Symbol	Value
Number of the equivalent array element	*N*	10
Wavelength of the transmitted signal	λ	0.2 m
Number of pulses in a CPI	*K*	12
Sampling rate of the base-band	*f_s_*	60 MHz
Pulse repetition frequency (PRF)	*f_r_*	2 kHz
Minimum slant-distance between target and radar	*R*_0_	10 km
Altitude for airborne	H	3 km
Speed of airborne	*V_a_*	100 m/s
The spacing of array elements	*d*	0.1 m
Noise variance	σ^2^	1
Normalized spatial frequency	${\bar{f}}_{s}$	0
Normalized Doppler frequency	${\bar{f}}_{d}$	0.3

**Table 2:** Interference Target Location and INR.

The Number of Interference	1	2	3	4	5	6	7	8	9	10
The number of range resolution cell	41	46	51	56	61	81	86	91	96	101
INR/dB	22	26	12	24	18	14	10	28	16	30

## Data Availability

Not applicable.
